# High Prevalence of Primary Multidrug Resistant Tuberculosis in Persons with No Known Risk Factors

**DOI:** 10.1371/journal.pone.0026276

**Published:** 2011-10-27

**Authors:** Larissa Otero, Fiorella Krapp, Cristina Tomatis, Carlos Zamudio, Francine Matthys, Eduardo Gotuzzo, Patrick Van der Stuyft, Carlos Seas

**Affiliations:** 1 Instituto de Medicina Tropical Alexander von Humboldt, Universidad Peruana Cayetano Heredia, Lima, Peru; 2 Epidemiology and Disease Control Section, Department of Public Health, Institute of Tropical Medicine, Antwerp, Belgium; Institut Pasteur de Lille, France

## Abstract

**Introduction:**

In high multidrug resistant (MDR) tuberculosis (TB) prevalence areas, drug susceptibility testing (DST) at diagnosis is recommended for patients with risk factors for MDR. However, this approach might miss a substantial proportion of MDR-TB in the general population. We studied primary MDR in patients considered to be at low risk of MDR-TB in Lima, Peru.

**Methods:**

We enrolled new sputum smear-positive TB patients who did not report any MDR-TB risk factor: known exposure to a TB patient whose treatment failed or who died or who was known to have MDR-TB; immunosuppressive co-morbidities, ex prison inmates; prison and health care workers; and alcohol or drug abuse. A structured questionnaire was applied to all enrolled participants to confirm the absence of these factors and thus minimize underreporting. Sputum from all participants was cultured on Löwenstein-Jensen media and DST for first line drugs was performed using the 7H10 agar method.

**Results:**

Of 875 participants with complete data, 23.2% (203) had risk factors for MDR-TB elicited after enrolment. Among the group with no reported risk factors who had a positive culture, we found a 6.3% (95%CI 4.4–8.3) (37/584) rate of MDR-TB. In this group no epidemiological characteristics were associated with MDR-TB. Thus, in this group, multidrug resistance occurred in patients with no identifiable risk factors.

**Conclusions:**

We found a high rate of primary MDR-TB in a general population with no identifiable risk factors for MDR-TB. This suggests that in a high endemic area targeting patients for MDR-TB based on the presence of risk factors is an insufficient intervention.

## Introduction

Drug resistant (DR) tuberculosis (TB) rates are increasing globally. Access to culture and drug susceptibility tests (DST) for diagnosis remains unavailable in many settings [1], [2]. Tailored case detection strategies for DR TB are required to maximize diagnostic efficiency and the rational use of financial, human and infrastructure resources. The objective of this approach is early detection of DR patients which in turn should allow early initiation of appropriate TB treatment regimens with prevention of clinical deterioration and reduction of transmission of DR strains.

Current international guidelines strongly recommend testing for DR in specific cases such as TB treatment failure, exposure to a multidrug resistant (MDR) case, return after default, relapse, in the presence of a co-morbidity, previous TB treatment with poor quality drugs or provided by a poor quality programme, and persistence of a positive smear at month two or three of a standard short course treatment [3]. In addition, DR testing is recommended in certain populations: HIV-positive patients in areas where HIV is associated with DR, prisoners, and in persons living in areas with high DR prevalence [3], [4].

In Peru, DST has been centralized for many years in the national reference laboratory. National surveys from 1996 and 2006 show an increase of MDR rates from 2.4% to 5.3% among new cases, and from 15.7% to 23.6% in previously treated cases, respectively [5], [6]. MDR rates are heterogeneously distributed within the country. Urban areas are most affected, with 58% of all TB cases and 82% of MDR-TB cases reported in Lima [7]. Also within Lima, a city of 9 million inhabitants, TB and MDR-TB burden is heterogeneously distributed among the districts. In most regions, DST is restricted to those with a higher MDR pre test probability [8]. This targeted testing has shown good results: in two districts of Lima, 34% of patients with at least one risk factor for MDR had indeed MDR [9]. A good selection of patients to be tested based on their pre test probability is a component of an efficient diagnostic process. In order to contribute with evidence for enhanced MDR case detection strategies, we aimed to evaluate the yield of testing low risk patients living in a high MDR burden area.

## Materials and Methods

### Study setting

The study was conducted in a semi-urban district in Lima with a population over one million inhabitants [10]. The district has a TB incidence of 213 per 100,000 inhabitants [11] and a MDR prevalence of 7% among all TB cases in the area [12]. Social security and private health care facilities also operate in the district. However, most TB cases are managed in the public health services [13]. The HIV prevalence in TB patients in this setting is similar to the national prevalence, which in 2008 was 2.6% [14].

Patient recruitment was conducted in all 34 public health care facilities (one hospital and 33 first level health care facilities) of the district. The NTP has designated offices in every facility. Following national guidelines, TB suspects with at least one sputum sample positive for acid fast bacilli are started immediately on TB treatment. Sputum culture and DST for first line drugs are routinely requested for any retreatment category (failure, return after default, relapse); persons reporting exposure to a MDR-TB case or to a TB case that failed treatment or that died during treatment; patients with immunosuppressive co-morbidities such as HIV and diabetes; persons working or admitted in a prison; health care workers, and persons with a recent and prolonged admission to a hospital [8]. Sputum samples from new TB patients who do not report one of these high risk factors for MDR are not cultured routinely. These patients start a regimen for drug susceptible TB which consists of two months of isoniazid, rifampicin, ethambutol and pyrazinamide taken six days per week followed by four months of isoniazid and rifampicin taken two days per week. During follow-up, patients are evaluated monthly for smear conversion; if smears remain positive at month two or three, a sputum sample is sent for culture and DST. A MDR consultation committee decides further management, usually starting a standardized second line regimen until full DST results are available for individualized regimens.

### Patient recruitment

From April 2008 to March 2010, adults diagnosed in the 34 health facilities with a first episode of smear positive TB and no reported risk factors for MDR-TB were eligible and invited to participate in the study. Trained field workers covered from Monday to Saturday from 8 am until 2 pm the facilities with large numbers of TB patients and twice or thrice a week those with fewer patients. They first screened eligible patients. We considered risk factors for MDR-TB all the criteria that the national guidelines use to request a culture and a DST among new smear positive cases, as well as alcohol and drug abuse. Patients reporting at least one of these factors were not enrolled. To correct for underreporting during screening we subsequently looked into more detail for these risk factors, and previous TB treatment, with a structured questionnaire applied to every participant enrolled. If a risk factor was still elicited in enrolled participants, all study procedures would be continued but these participants would be excluded in the analysis. Demographic data, exposure to TB, and clinical data such as use of previous prophylaxis was recorded for participants. Field workers instructed the patient on submitting a sputum sample. Patients submitting samples after taking more than three doses of anti tuberculosis treatment were also excluded.

If the patient had been recently tested for HIV, the result was retrieved from the clinical files. The NTP recommends and offers HIV testing to all TB cases. When the patient had not been tested recently, the field workers offered the patient the option to be screened. HIV positive patients were referred to the national HIV programme for management. HAART is provided free of charge to patients. Pre and post test counselling are done by qualified staff from the Ministry of Health.

### Sample collection and processing

If the patient could not produce a sputum sample on the spot, a morning sample was collected the next day. Sputa were transported within six hours to the microbiology laboratory at the Instituto de Medicina Tropical Alexander von Humboldt. Samples were kept at room temperature and processed on the day of arrival. If a sample arrived in the afternoon or on a Saturday, it was kept at 4°C until the next day or until Monday. A Ziehl-Neelsen smear was done, and the sputum was cultured in two slopes of Löwenstein-Jensen media. Positive cultures were tested for drug susceptibility using the 7H10 agar method and growth with the following drug concentrations of antibiotics defined resistance: 0.2 µg/ml isoniazid, 40 µg/ml rifampicin, 6 µg/ml ethambutol mg and 2 µg/ml streptomycin.

A sample of 74 mycobacterial isolates was sent for external quality control of DST to the Institute of Tropical Medicine in Antwerp, Belgium, a WHO/IUATLD supranational reference laboratory.

### Statistical analysis

Data were entered in Microsoft Access database (Microsoft, Redmond, WA, USA), and analyzed with EpiInfo version 3.5.1 (CDC, Atlanta, GA, USA). A TB patient was the unit of analysis and the DR pattern the outcome variable. The definition of a MDR case was resistance to isoniazid and to rifampicin on at least one Löwenstein-Jensen slope. “MDR-plus” was defined as MDR and resistance to another drug, “mono resistance” was defined as resistance to a single drug out of the four tested, “poly resistance” was defined as resistance to at least two drugs not involving the combination of isoniazid and rifampicin. The MDR rate was the proportion of MDR-TB cases among the patients that had a DST result.

Proportions in subgroups were compared using Pearson's Chi-square test; odds ratios (OR) were calculated with 95% confidence intervals (95% CI).

### Ethical considerations

This study was approved by the Institutional Ethics Committee at Universidad Peruana Cayetano Heredia and by the Regional Health Direction from the Ministry of Health. Written informed consent was obtained from all participants. All data were processed anonymously. A separate informed consent was used if the patient was to be tested for HIV.

## Results

**Figure 1:**
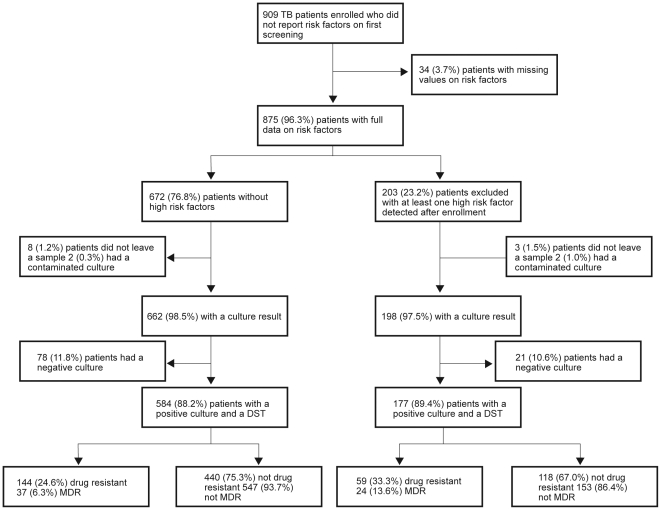
Patient enrolment and drug resistance results. Legend: DST = Drug susceptibility test; Drug resistant = any drug resistance, including MDR; MDR = multidrug resistant.

We enrolled 909 patients with a first episode of smear positive pulmonary TB that did not report a high risk factor for MDR-TB during screening. After thorough questioning, 203 did report at least one high risk factor for MDR-TB not detected initially and 34 did not give full information. Out of those enrolled but at known risk for MDR, 198/203 (97.5%) had a culture result available of which 177/198 (89.4%) were positive. MDR rate among this group was 13.6% (24/177). None of the participants with incomplete information on risk factors had MDR-TB. From the 672 not reporting a risk factor 13.1% (88) were excluded as they did not have a positive culture. Finally, among participants not reporting a risk factor that had a positive culture, 6.3% (95% confidence intervals (95%CI) 4.4–8.3) (37/584) were MDR. Enrolment process and MDR rates are shown in figure 1.

Of the isolates sent for DST external quality control, it was possible to culture 59/74 (79.7%) again. Concordance for isoniazid and rifampicin resistance profiles was 100% and 93.1% for ethambutol.

**Figure 2:**
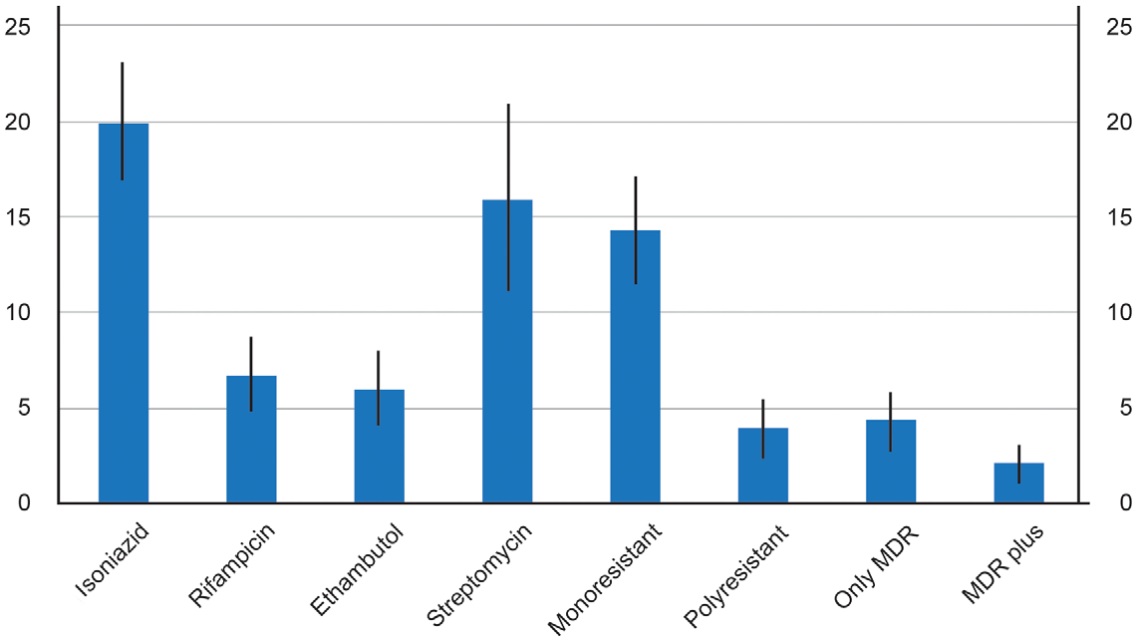
DST patterns in patients with no reported risk factors for MDR-TB: rates and confidence intervals. Legend: Mono resistant: resistance to a single drug out of the four tested; Poly resistant: resistance to at least two drugs not involving the combination of isoniazid and rifampicin; Only MDR: resistance to isoniazid and rifampicin only; MDR plus: resistance to isoniazid, rifampicin and another drug.

DST patterns for the low risk population are shown in figure 2. Overall, 117/584 (20.0%, 95%CI 16.8–23.3) were resistant to isoniazid and among the non MDR patients 80/547 (14.6%, 95%CI 11.7–17.6) were resistant to isoniazid, either mono or poly resistant.

Among the 584 patients at low risk of MDR, 391 were HIV negative and 193 had an unknown status. The MDR-TB rates in both groups were similar: 6.7% (95%CI 4.2–9.1) (26/391) and 5.7% (95%CI 2.4–9.0) (11/193), respectively. Table 1 shows the patient's characteristics associated with MDR-TB without known risk factors compared to those with either pan susceptible or with DR patterns other than MDR. We found no significant factors associated to MDR. Out of fourteen patients that reported previous use of isoniazid prophylaxis, none were MDR, six were resistant to isoniazid (five were mono resistant and one was poly resistant), and the other eight were pan susceptible.

**Table 1:** Characteristics and MDR status of patients without known risk factors.

Exposure variable		MDR	Non MDR	Crude OR (95% CI)
	N	n (%)	n (%)	
**Age**				
Median (IQR)		26.02 (23.16–32.04)	27.52 (22.58–36.56)	
<25	226	16 (7.1)	210 (92.9)	1
25–34	197	13 (6.6)	184 (93.4)	1.08 (0.51–2.30)
>35	159	8 (5.0)	151 (95.0)	1.44 (0.60–3.45)
**Sex**				
Male	308	19 (6.2)	289 (93.8)	
Female	268	18 (6.7)	250 (93.3)	1·09 (0.56–2.13)
**TB contact** *				
No	337	20 (5.9)	317 (94.1)	
Yes	247	16 (6.5)	231 (93.5)	1.09 (0.56–2.16)
**Previous prophylaxis**				
No	535	34 (6.4)	501 (93.6)	
Yes	14	0 (0)	14 (100.0)	0.5 (0.003–0.89)**
**Tobacco use**				
No	486	30 (6.2)	456 (93.8)	
Yes	79	7 (8.9)	72 (91.1)	1.48 (0.63–3.49)
**Residency within the district**				
Upper area	312	16 (5.1)	297 (94.9)	
Lower area	272	21 (7.7)	251 (92.3)	1.55 (0.79–3.04)

*Patient states having been in contact with a person with active TB who is alive and whose episode of TB was not report to be MDR TB.

**To calculate this odds ratio (OR), 0·5 was added to each value, and the standard formulae for OR, confidence intervals and p-value were applied.

## Discussion

High MDR rates were found among new smear positive pulmonary TB patients without known risk factors for MDR. This finding suggests that patients are at risk of infection with a MDR strain by living in a high incidence area where ongoing transmission takes place. The high burden of MDR shown in our data from an urban population may be masked in national surveillance or in national surveys of drug resistance based on a representative sample of TB patients in the country, when areas of low MDR rates predominate. These results should prompt further investigations in other districts of Lima and regions of Peru. The course of action derived from this study should be universal access to DST for populations living in districts with high incidence of MDR TB.

The study has some limitations. Only patients attending public sector health care facilities were included. However, in Peru, most cases of TB are treated within this sector [13]. Also, the selection of patients without risk factors relied on patient's report, which could have masked some risk factors. In particular, patients may not have reported previous TB treatment which might overestimate the rate of MDR among new cases. To limit under reporting, once enrolled, exhaustive probing for each factor was done. Finally, we might have lacked power to detect association with weak risk factors such as isoniazid prophylaxis. Nevertheless we have a representative population from a highly populated district as all public health care facilities were included. The negative culture results in a group (11%) of enrolled patients might have been caused by sterilization after taking one or two doses of rifampicin, or by a failure in the transport or decontamination procedures set for the study [15].

The risk factors associated with primary MDR-TB from other studies cannot be compared to our results as patients with known risk factors were excluded. We did not find any additional variable associated with primary MDR-TB. TB treatment failure is the strongest factor associated to MDR [9], [16]–[19] followed by exposure to an MDR-TB case or to a case failing treatment. Female sex has been associated with MDR-TB in some settings [18], [19] as well as lung cavities [20], [21].

Enrolled patients with risk factors had a high rate of primary MDR (13.6%). This rate is most probably underestimated as this study only enrolled patients with high risk factors that were not reported during initial screening. The cost effectiveness of testing all TB cases in high MDR burden areas and only high risk groups in low risk areas for MDR TB should be evaluated. The feasibility of testing large numbers of patients, the tests to be used, and the impact on the efficiency of local, regional and reference laboratories should also be considered. Introduction of expensive rapid methods should probably be restricted to settings where their positive predictive value will be high. Except when extremely specific, they will yield a high proportion of false positives in areas with low MDR prevalence and may not be a cost effective as a screening tool.

The high proportion of primary MDR found among patients without risk factors attending primary health care facilities in a populated district in Lima suggests transmission of drug resistant strains among the general population. This indicates a need for scaling up strategies to reduce transmission of MDR TB: early detection, proper management of MDR TB cases, infection control measures, and addressing the high defaulter rates from both sensitive and resistant TB. This study suggests that in a high endemic area targeting patients for DST based on the presence of risk factors is clearly an insufficient intervention for early case detection of MDR TB.
